# Patients’ expectations of acute low back pain management: implications for evidence uptake

**DOI:** 10.1186/1471-2296-14-7

**Published:** 2013-01-08

**Authors:** Tammy C Hoffmann, Chris B Del Mar, Jenny Strong, Juliana Mai

**Affiliations:** 1 Associate Professor of Clinical Epidemiology, Centre for Research in Evidence-Based Practice, Faculty of Health Sciences and Medicine, Bond University, Gold Coast, Queensland 4229, Australia; 2 Division of Occupational Therapy, School of Health and Rehabilitation Sciences, The University of Queensland, Brisbane, Queensland, Australia; 3 Centre for Research in Evidence-Based Practice, Faculty of Health Sciences and Medicine, Bond University, Gold Coast, Queensland, Australia

**Keywords:** Low back pain, Doctor-patient communication, Patient expectations, Clinical practice guidelines, Primary health care

## Abstract

**Background:**

In many countries, general practitioner (GP) care of acute low back pain often does not adhere to evidence-based clinical guidelines. There has been little exploration of this deviation from evidence-based care from the patients’ perspective, particularly in relation to patients’ care expectations. The aim of this study was to explore the care expectations in patients who present to their GP with acute low back pain, influences on expectation development, and congruence of these expectations with clinical guideline recommendations.

**Methods:**

Qualitative study in an inner urban general practice in Brisbane, Australia. Semi-structured interviews were conducted with 11 patients who presented to their GP with acute low back pain.

**Results:**

Patients had a biomechanical understanding of back pain, how it should be tested and treated, and a poor understanding of its natural history. Most expected x-rays, believing they were necessary to identify the “cause of the pain” without belief of any downsides to x-rays. Patients’ expectations were primarily influenced by the experiences of family and friends, their own previous experiences of low back pain care, and comments from other health professionals they were consulting. The GP-patient relationship was important in influencing patient satisfaction of care provided. Most patient expectations, and some of the care that they reported receiving, were incongruent with guideline recommendations.

**Conclusions:**

A biomechanical approach to management rather than an awareness of empirical evidence was evident in patients’ expectations. Communication and education by the GP that includes specifically enquiring about patients’ expectations, provides an opportunity to correct misperceptions, explain acute low back pain natural history, and the rationale for test and treatment recommendations.

## Background

Low back pain consultations are among one of the most common reasons for general practice appointments in many developed countries [1,2]. Key recommendations in evidence-based clinical practice guidelines include patients staying active and avoiding bed rest, trying paracetamol as the first choice of analgesic, and general practitioners (GPs) providing education, reassurance of likely positive prognosis, and not routinely ordering radiological examinations unless ‘red flags’ are identified [2-4]. Although patients’ health will benefit from their clinicians’ adherence to these guidelines, in many countries, usual GP care of acute low back pain does not typically adhere to guidelines [5].

Various reasons for non-adherence to guidelines have been reported. [6-8] In addition to clinician-related barriers such as knowledge and beliefs, patients are themselves a powerful influence on GPs’ behaviour [8,9]. GPs report accommodating patient demands, such as for x-rays [9] and that the care they provide is sometimes influenced by very indirect information, such as *their* perceptions of patients’ expectations [8].

The problem of GPs’ deviation from evidence-based care from the patient perspective in relation to patients’ actual expectations has been insufficiently explored. We aimed to explore the expectations of the management of patients presenting to primary care with acute low back pain, influences on the development of these expectations, and their congruence with guideline recommendations.

## Methods

This study received ethical approval from the Behavioural and Social Sciences Ethical Review Committee of The University of Queensland.

### Design

We chose a qualitative design using a descriptive phenomenological approach [10,11] and purposive sampling and interviews to enable a clearer understanding of patients’ perspectives in the consultation experience.

### Participants

Patients were eligible for inclusion if they: were 18 years or older; presented to their GP with an episode of acute low back pain (defined as an episode of pain present for less than three months [2]); and had adequate English, cognition and communication "as determined by the treating GP" to provide written informed consent and participate in a telephone interview.

### Procedure

A convenience sample of patients was recruited from an inner urban general practice in Brisbane, Australia, between April and July 2011. The medical centre is located in a suburb with a bimodal socio-economic profile, with large proportions of both high income and low income individuals [12]. Patients can typically see their usual GP if appointments are made at least one day in advance. All 10 GPs working there were invited to recruit all eligible patients during the study timeframe; participants were recruited by four GPs. In the consultation, the GP explained the study and obtained patients’ consent to provide their contact details to the research team. For those who agreed, further written information was provided by the medical centre staff, written informed consent obtained, and their telephone number provided to the research team. A convenient time for a telephone interview was scheduled.

### Data collection

One of the authors (JM) completed a semi-structured telephone interview with each patient. Interviews were audio-recorded, with an average duration of interviews of 20 minutes. An interview guide was used, based on research on barriers to evidence-based practice from the patient perspective, including barriers to guideline implementation. The guide focused on topics such as patients’ expectations of testing and treatment procedures for low back pain and how these expectations were conceived. The interview guide (available from the corresponding author on request) was used flexibly so that the interviewer could explore patients’ comments further if needed. Data saturation was reached when no new ideas or constructs arose in two consecutive interviews [10,13].

### Data analysis

Each interview was transcribed verbatim, using pseudonyms, prior to inductive thematic content analysis [14]. Two of the authors (JS, JM) independently read and re-read the transcripts and identified themes using colour coding, Post-it notes and annotation. In a face to face meeting, they discussed the themes until consensus was reached. A third author (TH) then coded the transcripts using the extracted themes, and was able to allocate text chunks to the aforementioned themes. The themes and illustrative quotes were then agreed to by all authors. This investigator triangulation added to the rigor of the study. Three of the authors are experienced evidence-based practice academics, and thus had particular views on the importance of using clinical guidelines to guide clinical practice. The fourth author (JM) was a research student whose opinions were less well shaped. She interviewed all patients, to ensure both consistency and bracketing of any bias.

## Results

Data saturation was reached when no new themes were identified in two consecutive interviews at interview 11, completing the recruitment phase. Of three additional patients invited to participate in the study, two declined (one too busy to participate and one not interested in being interviewed), and one was excluded because the primary reason for her consultation was not acute low back pain. Ten women and one man, with an average age of 52 years (SD =57, range 22 to 72) were interviewed. Three of the participants had work-related acute low back pain. Table 1 lists participants’ demographic characteristics.

**Table 1:** Participant demographics

**Study ID**	**Age (years)**	**Gender**	**First episode of back pain**	**Saw usual GP**	**Occupation**	**Highest level of education completed**
1	22	Female	Yes	Yes	Student and receptionist	TAFE^a^
2	69	Female	No	Yes	Retired	High school
3	60	Female	No	No	Office worker	High school
4	48	Female	No	Yes	Office worker	University
5	69	Female	No	Yes	Retired registered nurse	TAFE
6	33	Female	Yes	No	Restaurant manager; group fitness instructor	TAFE
7	46	Female	Yes	No	University lecturer	University
8	72	Male	No	Yes	Retired	High school
9	37	Female	No	Yes	Fitness instructor	High school
10	51	Female	No	Yes	Office management	University
11	67	Female	Yes	No	Retired	High school

^a^A Technical and Further Education (TAFE) Institute -offers predominately vocational tertiary education courses.

The themes which emerged could be framed around three areas: patient expectations about the testing and treatment arising during their consultation; influences on patients’ expectations; and the importance of the relationship between the patient and the GP. Table 2 contains the themes and illustrative quotations.

**Table 2:** Themes and illustrative quotes

**Quote letter (used in text)**	**Illustrative quote (participant ID in brackets)**
**Patients’ expectations of tests and beliefs about x-rays**	
**A**	*Because it … was pretty bad, so I figured… need [an x-ray] to see what it was...They [x-rays] can isolate the problem* (P1)
**B**	*I thought it [x-ray] might show the cause in my spine. I think it helps* (P4)
**C**	*I guess [an x-ray] was to establish whether, from my perspective, whether it was just a pulled muscle or whether it was called herniated disc or whatever.* (P7)
**D**	*I knew there was something wrong and I thought well, I was just guessing and without actually seeing* (P8)
**E**	…*gave me a physical examination and she said well clearly there was a problem and maybe we should confirm her findings with an x-ray…* (P5)
**F**	*…the whole thing sort of confirmed what we knew, that I had wear and tear* (P11)
**G**	*… to find out what it is and try to fix it* (P7)
**H**	*No, just positive reassurance I think*. (P10)
**I**	*I think they’re like the last resort because it’s like radiation on your body, like if it’s not necessary and plus it’s the hours of your whole day that you’ve got to take out to get it done and wait.* (P1)
**J**	*The only downside maybe … was the radiation, but that was the only negative. I think the less radiation you can be exposed to the better, but that having being said the results justified that risk. I would rely on my doctor’s advice.* (P8)
**Patients’ treatment expectations**	
**K**	*I had no expectations.* (P5 and P8)
**L**	*I thought she’d say go see a physiotherapist, and she was going to give me a letter yesterday but she said she’ll get the results of the x-ray first.* (P2)
**M**	*I didn’t think that a doctor could do anything apart from give me pain relief as in medication* (P3)
**N**	*I had no idea, so I guess, a painkiller obviously. The other thing, um the anti-inflammatory, I guess I didn’t expect an anti-inflammatory but it worked really well* (P7)
**O**	*I think [the GP] should always try to find out what the cause is and take steps to eliminate that if possible, even if it’s just a simple exercise or even if it’s just lying down, I think it’s important that they try and find a way of helping the situation.* (P4)
**P**	*Because if you don’t get something treated it can impact on other parts of your body.* (P9)
**Q**	*I kind of felt either I’d left it too long and it’d got to the stage where it just needed something.* (P10)
**R**	*It depends, I suppose. In my case it was quite severe and I had, there was no option but to get something done.* (P8)
**Influences on patients’ expectations**	
**S**	*I’d heard about friends having MRIs and the osteopath suggested that I have an MRI to look at the soft tissue, and [the doctor] suggested the x-rays and I did have the x-rays and the verdict is that there is wear and tear* (P11)
**T**	*[The chiropractor] said to me ‘Before I touch you I want you to get an x-ray done.’* (P10)
**U**	*Because that’s what’s happened before* (P8)
**V**	*Probably because I’d never had one [x-ray]done, and that’s the usual, the doctor probably wants to see what’s happening.* (P4)
**W**	*Physio wasn’t working…it wasn’t getting better… and I knew that I needed to sort of have something further checked out* (P11)
**X**	*Well in most cases that I’m aware of your doctors just tend to put a patient on anti-inflammatories and away you go.* (P9)
**Y**	*I assumed I probably would get some sort of anti-inflammatories… because it was a muscle…* (P5)
**Z**	*Anti-inflammatories… aren’t they like strong antibiotics that will deal with the infection?* (P11)
**Importance of the GP-patient relationship**	
**AA**	*I suppose it’s like any clinical part of what a doctor does, it’s the experience, the amount of patients they see, that they find the tried and true methods and that’s what happened.* (P5)
**BB**	*I went and saw my doctor again because I was happy with past results and she knew my history* (P8)
**CC**	*I’m sure he’s got guides as to what to use when, so, and experience.* (P7)
**DD**	*Yeah, a lot depends on sort of your relationship with your GP. I think in this case the doctor’s advice is good* (P10)
**EE**	*I knew she would zero in on what the problem was… I was quite happy to leave it in her hands. I would rely on my doctor’s advice* (P7)
**FF**	*[My doctor] has been treating me for quite a long time, and she has been extremely proficient at diagnosing things I have, and… she knew what she was doing. She had come across this before… and immediately zeroed in on the problem. Her experience was a factor, and my experience with her as a doctor immediately made me go straight to her and say look, I need help here.* (P8)

### Patient expectations

#### Test expectations

Most patients expected their GP to refer them for an x-ray, particularly patients who felt that their pain was severe (Table 2, quote A). Many patients believed that an x-ray would enable the cause of the pain to be determined (quote B), and that the problem would be able to be seen (quote C). Patients felt that x-rays played an important role in providing reassurance as well as confirmation of their GPs’ diagnosis (quotes D-F). Many patients believed that by identifying the cause of their pain through x-ray, the GP would then know how to ‘fix it’ (quote G). Very few patients expected a physical examination to be performed and a few patients had no expectations about what the tests their GP would perform/request.

Some patients reported receiving the testing procedures they had anticipated (namely, an x-ray referral), while some stated their GP provided procedures they had not expected to receive (including a physical examination). Patients reported satisfaction with their GP’s care, apart from one who wanted to have an MRI and was given an x-ray referral instead.

Most patients, including both those that did and did not have an expectation of an x-ray, believed that there were no downsides associated with x-rays (quote H). A few expressed minor concerns about radiation and time inconveniences. However, these patients reported that the usefulness of an x-ray outweighed these potential negatives (quotes I, J), assuming that their GP had already balanced these benefits and harms.

#### Treatment expectations

There was variation among patients regarding the treatments they were expecting from their GP. Many did not know what to expect (quote K); some expected a referral to another health professional (e.g. physiotherapist) (quote L); others expected analgesics (quotes M, N). When asked about the option of ‘no treatment’ for low back pain with the exception of analgesics, most believed in a biomechanical approach of needing to find the problem and fix it in a timely manner (quotes O-Q). A few patients felt that no treatment may be suitable for some people, but not for them (quote R).

Patients were advised to use, or prescribed, anti-inflammatory drugs and analgesics. Some patients reported being told to avoid vigorous activities; others were told to stay active, including specific muscle strengthening exercises. Other treatments that patients reported receiving from their GP were referrals to allied health professionals, and recommendations for orthotics. There was no expressed dissatisfaction with GP treatment.

### Influences on patients’ expectations

Patients’ expectations about diagnostic investigations had been influenced by family, friends and/or other health professionals (particularly osteopaths and chiropractors) (quotes S, T). Prior experiences of care for low back pain were an influence for those who had experienced it before (quote U). However many patients could identify no specific influences on their expectations, instead reiterating their biomechanical model. Those who expected an x-ray assumed it was part of standard procedure (quote V) and those experiencing no improvement expected a change in management (quote W). Of the patients who had articulated particular treatment expectations, these were predominantly influenced by prior experiences of family and friends (quote X). When patients held a biomechanical model, the assumed cause of their low back pain determined their treatment expectations (quotes Y, Z).

### Importance of the GP-patient relationship

When asked how their GP knew what management to adopt, most patients identified their experience (quote AA). Some thought care was guided by the GP’s knowledge of the patient and his/her medical history (quote BB). One patient mentioned a guide, although it is not clear whether this referred to a research-based guide (quote CC). Patients’ level of trust and confidence in their GP, particularly in patients who had seen their usual GP, influenced their overall satisfaction about the consultation, and confidence in their GP’s management (quotes DD-EE). Those reporting discrepancies between their expectations of care and that actually provided by the GP, described satisfaction with the consultation if they had a strong, and previously successful, relationship with the GP (quotes FF).

## Discussion

### Summary of main findings

Key findings were that patient expectations included: almost universally that x-rays were standard; a wide variety of treatments; a biomechanical understanding was necessary to dictate management; and a naivety about contemporary empirical clinical evidence on best practice for acute low back pain.

Patients in this study expressed a number of misperceptions including that severity of the pain is an important indicator and that the cause of the pain needs to be identified and is necessary for guiding treatment. Any (even incidental) findings on x-ray were thought to indicate the cause of the pain. Patients were referred for x-ray, despite guideline recommendations that this is unnecessary for most in primary care. In Australia, 25% of acute low back pain primary care patients are referred for imaging [5]. Patients were not aware of the limited diagnostic value of x-rays for acute low back pain. Nor did most consider x-rays to have drawbacks, or, at least assuming that if they did, the benefits outweighed the risks. Patients assumed that clinicians will only make recommendations such as ordering x-rays if they believed them necessary, safe and effective.

Patients felt that their back pain needed to receive active treatment, particularly to prevent it from getting worse. They demonstrated little understanding of its natural prognosis. A key guideline recommendation is that patients should receive education and reassurance [2], yet patients did not perceive this as sufficient, wanting “something done” in addition. Patients expected “painkillers” rather than paracetamol when this is the recommended first choice of analgesic [2], perhaps helping to explain why Australian GPs recommend non-steroidal anti-inflammatory drugs (NSAIDs) most often for acute low back pain [5]. Patients commonly saw “painkillers” and anti-inflammatory drugs as having distinct actions, without appreciating the overlap.

### Strengths and limitations of the study

Our study approached a well-known problem from a perspective that has been rarely studied. It provides insights into patients’ management expectations when consulting a GP for acute low back pain. These insights can be used to guide the interaction that GPs have with patients with acute low back pain. We did not recruit a random sample of participants as is appropriate for qualitative research, but our sample was a convenience sample, relatively small, with an unequal gender distribution, and participants from only one medical centre. However, our study is strengthened by including patients from a range of ages, occupations, and education levels, and consulting either their usual GP or another. Self-reported patient recall imposes some concerns about the accuracy of patients’ recall of events from the consultation and patients’ expectations may have been influenced as a result of the consultation.

### Comparison with existing literature

Patients were satisfied with their GP consultation, even when their management expectations were not met - possibly because of a strong doctor-patient relationship. This is fundamentally important in promoting patients’ trust [15], perhaps even more so when expectations are not met. That GPs are keen not to fail their patient’s expectations has been previously identified as a major barrier to implementing low back pain guidelines [8].

In facilitating patient-centred care and shared decision making, a fundamental step is for clinicians to elicit patients’ expectations; discuss, and correct any of their misperceptions, expectations or fears [16]. However patients in this study appeared to have been provided with little education about ordering, or not ordering, x-rays, their downsides, and the natural history of acute low back pain, including its likely favourable prognosis. As this was an exploratory study eliciting patients’ perspectives and we did not audit the care that patients actually received, it is possible that more education was provided than reported. Although if so, patients were not able to convey this information to the interviewer, possibly because the information was not understood, retained, or accepted by the patients.

Not unexpectedly, patients’ expectations were predominately influenced by their previous experiences of low back pain care and advice received from family, friends, colleagues and health professionals. But the messages from other health professionals were alarmingly inconsistent, as noted previously [8] and further support the need for clinicians to explore patients’ expectations early in the consultation so that misperceptions can be addressed. Patients in our study also lacked awareness that GPs should use the latest empirical research evidence to guide their practice.

### Implications for future research

Future enhancements to this area of research could include interviewing patients prior to the consultation, and recording the consultation so that the actual care received is captured to further explore the doctor-patient encounter.

## Conclusion

Patients’ biomechanical understanding of back pain, their subsequent test and treatment expectations, poor understanding of the natural history of low back pain, and message inconsistency from health professionals suggest a need for public education about the appropriate management of acute low back pain. A public education campaign that ran in the mass media in Victoria, Australia for 22 months improved back pain beliefs in both the general public and GPs [17], with the effects sustained 3 years after the campaign ended [18]. In the absence of such a campaign, GPs are encouraged to specifically enquire about patients’ expectations to correct common misperceptions, and better educate patients about the prognosis of acute low back pain, the role of imaging, and management recommendations with their rationale.

## Competing interests

The authors declared that they have no competing interest.

## Authors’ contributions

TH, JS, and CDM conceptualised the study. JM was responsible for data collection. JM, JS, and TH were responsible for most of the data analysis, with some input from CDM. All authors read and approved the final manuscript.

## Pre-publication history

The pre-publication history for this paper can be accessed here:

http://www.biomedcentral.com/1471-2296/14/7/prepub
